# P05.18. Mixed methods approaches in whole systems research: a study of Ayurvedic diagnostics

**DOI:** 10.1186/1472-6882-12-S1-P378

**Published:** 2012-06-12

**Authors:** A Dhruva, S Adler, J Weaver, M Acree, C Miaskowski, D Abrams, F Hecht

**Affiliations:** 1 University of California, San Francisco, San Francisco, USA

## Purpose

To investigate the clinical rationale and agreement between raters for making Ayurvedic diagnoses, using a mixed methods approach.

## Methods

Three 30 minute Ayurvedic assessments, including a history and limited physical exam, were videotaped and then viewed by Ayurvedic clinicians. The clinicians were asked to identify the primary and secondary dosha (Ayurvedic physiologic principle) involved in the patient’s prakruti (mind-body constitution) and vikruti (current state of the doshas) and explain their rationale for making a diagnosis. Cross-sectional comparison and thematic analytic approaches were used to analyze qualitative data.

## Results

Of 13 participants to date, 77% had at least a 6-year Bachelor of Ayurvedic Medicine and Surgery degree; participants had a mean of 15 years of clinical experience. Overall agreement on diagnoses ranged between 60-100% and was higher for vikruti (mean 86%) than prakruti (mean 75%). The mean rating of the similarity of the video format to real life was five (0-10 scale). Qualitative themes reported were: 1) Participants felt the video format limited their ability to ask about specific clinical information. 2) Participants explained that the video format was limiting because more clinical details obtained in a longer interview, an in-person physical exam, and re-evaluation of findings over time are important in making an accurate prakruti diagnosis.

## Conclusion

A moderate to high degree of agreement was found in the quantitative analysis and was better for vikruti than prakruti diagnoses. The video format, though well suited to the research paradigm, was limiting for the clinicians in terms of real world practice. Even when there was disagreement in diagnoses, the clinical rationale provided by clinicians was consistent with the theoretical basis of Ayurvedic medicine. A mixed methods approach was well suited to the study of Ayurveda as a whole system of medicine, but whole systems research remains challenging due to the complexity of the whole system.

